# Lightweight architecture optimization of YOLOv12n for improved cotton verticillium wilt detection

**DOI:** 10.3389/fpls.2026.1822081

**Published:** 2026-05-13

**Authors:** Zhuang Ye, Mi Yang, Ze Zhang, Zhenan Hou, Xinbo Zhao

**Affiliations:** 1 Xinjiang Production and Construction Crop Oasis Eco-Agriculture Key Laboratory, College of Agriculture, Shihezi University, Shihezi, China; 2 Key Laboratory of Tarim Oasis Agriculture, Ministry of Education, Tarim University, Alar, China

**Keywords:** channel aggregation block, cotton, detection, disease, ODConv, StarNet

## Abstract

To address the significant morphological variability of cotton Verticillium wilt lesions and the complex background interference present in field environments, existing detection models often struggle to achieve an effective balance between detection accuracy and model complexity. In this study, a precise and lightweight detection model, YOLO-SCOD, is proposed based on the YOLOv12n framework to enhance lesion recognition performance. During the feature extraction stage, YOLO-SCOD adopts the StarNet architecture as the backbone network. Its efficient feature mapping mechanism enhances the ability to interact with multi-scale features, thereby optimizing the model’s capacity to represent multi-scale lesion information. Meanwhile, a channel aggregation block is integrated into the C3k module of the neck network. Through adaptive channel reallocation and enhancement of key lesion features, the perception capability of the C3k2 and A2C2f modules for discriminative lesion features is improved. In the detection head, depthwise convolution is replaced with omni-dimensional dynamic convolution, which dynamically and adaptively adjusts convolutional weights through multi-dimensional attention collaboration, further improving the model’s localization accuracy and recognition capability. Experimental results demonstrate that the YOLO-SCOD model achieves improved performance improvements in the task of cotton Verticillium wilt detection. Compared with YOLOv12n, its precision and recall increase to 0.960 and 0.911, respectively, while mAP50–95 improves by 6.436%. In addition, the number of model parameters, FLOPs, and model size are reduced by 13.728%, 20.635%, and 12.727%, respectively, and inference speed increased by 4.167%. While maintaining high detection accuracy, YOLO-SCOD exhibits favorable lightweight characteristics, providing a viable solution for efficient automatic identification and intelligent detection of cotton Verticillium wilt.

## 1 Introduction

Cotton, as an important natural fiber raw material, is widely used in the textile industry (Ahmad and Hasanuzzaman, 2020; Gupta and Gupta, 2023). However, cotton Verticillium wilt, caused by the soil-borne fungus *Verticillium dahliae*, is widely distributed worldwide and often leads to yield reduction and deterioration of fiber quality; in severe cases, it can even result in plant death (Bardak et al., 2021; Li et al., 2025a; Zhu et al., 2023). Traditional diagnostic methods for cotton Verticillium wilt mainly rely on manual identification (Li et al., 2025b). Such approaches are not only highly subjective and inefficient but also difficult to meet the real-time detection requirements of large-scale farmland operations (Yang et al., 2024). For the long-term and stable advancement of the cotton industry, it is therefore essential to develop rapid and precise techniques for the detection and identification of Verticillium wilt.

Machine learning and computer vision have gradually alleviated the limitations of traditional cotton disease identification methods (Tan et al., 2026). Compared with conventional manual identification approaches, machine learning algorithms possess stronger data processing capabilities and can effectively discriminate disease images by constructing feature mapping models (Dinesh et al., 2025; Khan et al., 2025; Wani et al., 2022). Kumar et al. (2024) proposed a hybrid machine learning architecture for early-stage cotton disease identification, in which the prediction results of multiple models were integrated and a voting mechanism was introduced to obtain the final decision. The results of experiments showed a high level of recognition confidence for an ensemble model consisting of random forest and decision tree. Nainwal et al. (2025) comparatively evaluated the performance of nine algorithms, including logistic regression, support vector machines, and VGG-16, on soybean disease detection tasks. The results demonstrated the potential of machine learning methods for recognizing soybean aerial blight disease. However, machine learning algorithms tend to rely on manually designed features and are limited in their ability to generalize. When confronted with large-scale data and real-time detection requirements, they still struggle to meet the demands of modern intelligent agricultural applications (Janarthan et al., 2022; Yao et al., 2023).

Deep learning techniques, with their powerful feature self-learning capability, effectively overcome the dependence of traditional machine learning methods on manual feature extraction (Faisal et al., 2025; Hari and Singh, 2023). Using deep neural networks, complex multi-dimensional target features can be extracted directly from the original RGB image, thus significantly reducing the information loss that may occur in the process of manual feature design. This makes it possible to distinguish between healthy areas and diseased areas (Gao et al., 2025; Pan et al., 2024). At present, the target detection research based on deep learning mainly focuses on in-depth optimization of the basic model architecture, aiming to balance detection accuracy and computing efficiency, so as to enhance the robustness and practicality of the model in complex agricultural environments (Khalid and Talukder, 2025; Ouamane et al., 2025; Shafik et al., 2024). Tian et al. (2019) used densely connected neural networks to optimize the low-resolution feature layer in YOLOv3 in response to the detection of anthracnose lesion in the orchard environment, which effectively improved the detection performance and realized the real-time monitoring of the disease. Wang et al. (2025b) developed a deep learning–based approach to identify pepper diseases and insect infestations under greenhouse conditions. Building upon the YOLOv10 architecture, their method integrates an adaptive module for multi-scale feature extraction, a dynamic feature pyramid network, a small detection head, and the Inner-CIoU loss function. Experimental results demonstrated that, relative to the baseline YOLOv10n model, the proposed framework achieved an increase of 11.88% in mAP50 performance. Liu et al. (2025) enhanced the performance of a YOLOv11n-based model for cucumber disease detection by introducing deformable convolutional networks, an optimized small-object detection layer strategy, and a target-aware loss function. Zhao et al. (2022) developed an improved Faster R-CNN architecture for the recognition of diseases on strawberry leaves, flowers, and fruits. By integrating ResNet, a feature pyramid network, and CBAM blocks, the model achieved high recognition accuracy while maintaining satisfactory detection speed.

The manifestations and severity of Verticillium wilt vary significantly across different growth stages of cotton (Ayele et al., 2020; Cheng et al., 2025). In RGB images, cotton leaves infected with Verticillium wilt typically exhibit a color transition from green to yellow-brown, accompanied by gray spots, wilting, or desiccation. However, the cotton field operation environment is complex and changeable, which puts forward higher requirements for the robustness of the Verticillium wilt recognition algorithm (Li et al., 2024, 2025; Nie et al., 2024). In practical applications, factors such as leaf blocking each other, obvious plant stratification and serious background interference will adversely affect the accuracy and stability of disease detection (Zhang et al., 2022). The existing target detection technology still has the problem of insufficient detection accuracy in complex field environments. How to achieve an effective balance between model lightweight design and real-time performance is still a challenge (Aslam et al., 2025; Johri et al., 2024). In order to meet the above challenges, this study proposes a new model for the identification and detection of cotton Verticillium wilt, termed YOLO-SCOD, by optimizing the architecture of the YOLOv12 model. The proposed model aims to improve the model’s recognition performance under complex background conditions. The specific model optimization strategies are summarized as follows:

1. To address computational resource constraints in field environments, the StarNet architecture was integrated into the YOLOv12 backbone network. This approach reduces computational overhead while enhancing the model’s ability to extract features indicative of yellow wilt disease.
2. A channel aggregation block (CA block) is incorporated into the C3k module to construct the CA_C3k2 and CA_A2C2f modules, thereby reducing information loss during feature transmission and improving the integrity of feature representation.
3. To improve the model’s localization accuracy and scale adaptability for Verticillium wilt lesions, omni-dimensional dynamic convolution (ODConv) is added to the detection head.

## 2 Materials and methods

### 2.1 Data acquisition and preprocessing

**Figure 1:**
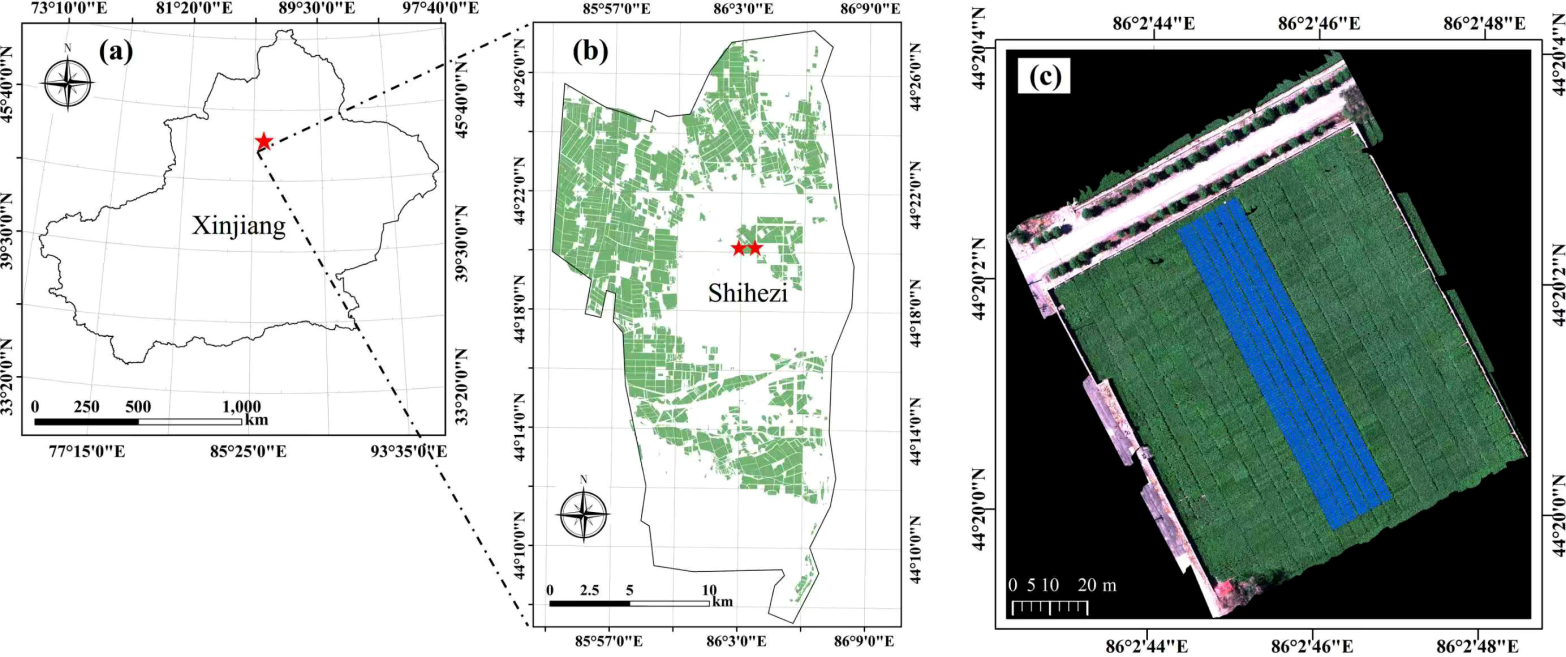
Geographical location and satellite image of the experimental disease field in Shihezi, Xinjiang. **(a)** Geographic location map of Xinjiang Province; **(b)** Geographic location map of Shihezi City in Xinjiang Province; **(c)** Satellite image of the experimental disease field.

The experimental area of this study is located at the cotton Verticillium wilt experimental field of the Academy of Agricultural Sciences, Shihezi City, Xinjiang Province (44.33°N, 86.05°E), and its geographical location is shown in **Figure 1**. This experimental field has long been maintained under relatively uniform Verticillium wilt inoculum conditions, providing a stable and controllable disease environment for the occurrence of cotton Verticillium wilt. The study area is situated on the northern foothills of the Tianshan Mountains and the southern margin of the Junggar Basin, characterized by a typical temperate continental climate. From August to September each year between 2021 and 2023, this study used an iPhone 13 to capture images of cotton plants infected with Verticillium wilt from various angles and heights, with the aim of constructing a multi-view and multi-scale RGB dataset for Verticillium wilt in cotton. After data cleaning and cropping, a total of 471 raw images with a resolution of 1024×1024 were collected. Subsequently, the data were manually annotated using Labelimg software, with the annotation class designated as “Cotton Verticillium Wilt”.

**Figure 2:**
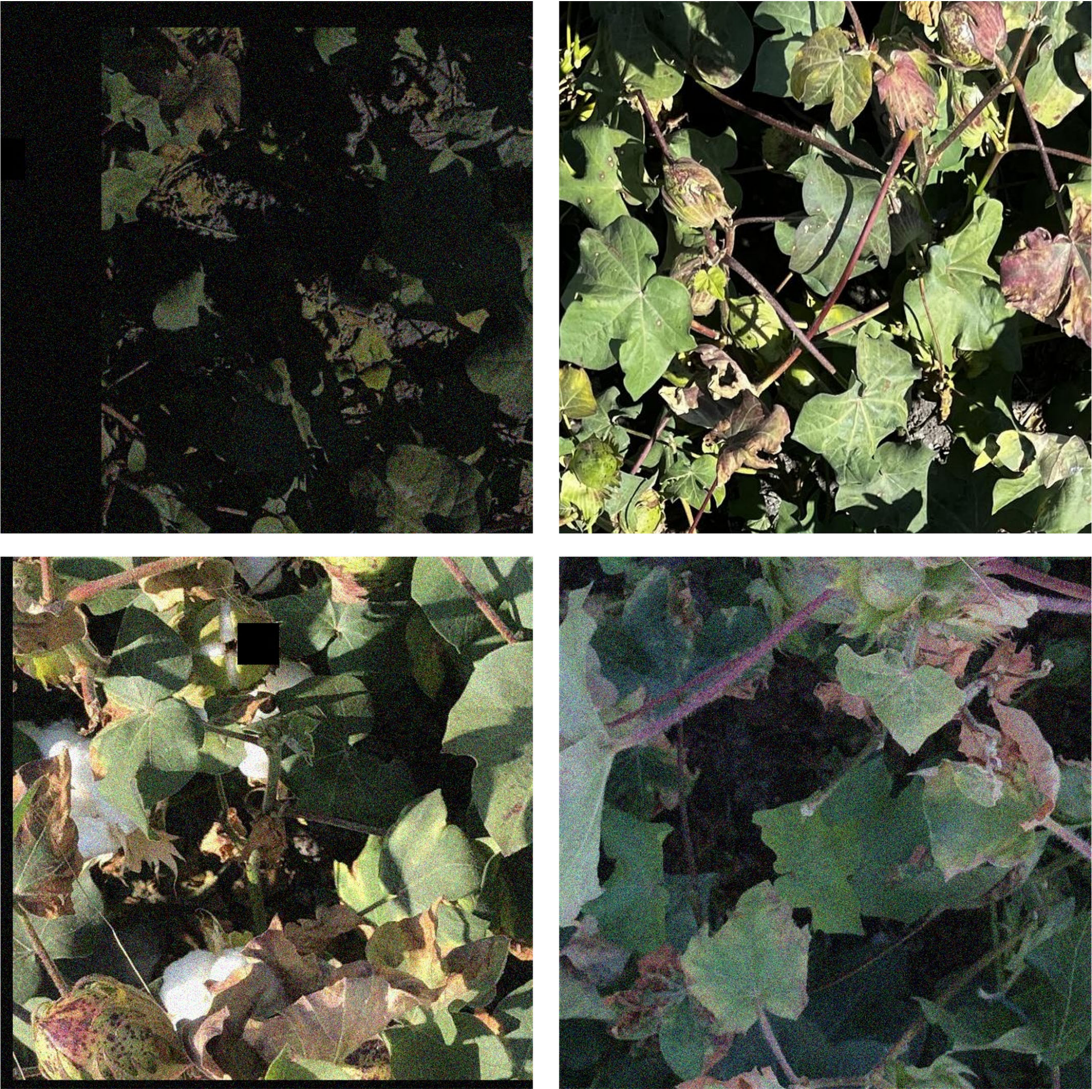
Examples of augmented images.

Data augmentation was used on the original cotton Verticillium wilt images to make the model more adaptable to complicated field situations. The augmentation strategies included geometric transformations, changes in lighting conditions, random noise addition and random local area occlusion. In order to reduce the sensitivity of the model to the blade posture and imaging angle, we use random flipping, angle rotation and translation to simulate the change of shooting angle and position. To cope with the frequent changes in lighting conditions in the natural environment, we randomly adjusted the light intensity to simulate the imaging differences under different weather conditions and light levels. In addition, random noise was added to the original images to simulate the interference factors in the acquisition process, thus improving the robustness of the model to noise interference. Random region occlusion was used to simulate phenomena such as leaf overlap and branch occlusion, enhancing the model’s ability to cope with locally missing information. By using these data augmentation methods, a total of 2355 images of Verticillium wilt in cotton were ultimately produced, containing 5785 annotated regions. This effectively enriched the feature diversity of the dataset and mitigated the risk of overfitting during model training. During model training, the augmented dataset was split into a training set (1886 images), a test set (235 images), and a validation set (234 images) in an 8:1:1 ratio. Examples of augmented images are shown in **Figure 2**.

### 2.2 Construction of the YOLO-SCOD detection model

#### 2.2.1 Overall structure of the YOLO-SCOD model

YOLOv12 improves both detection accuracy and efficiency at the same time by systematically optimizing feature representation and network architecture, while still retaining the benefits of end-to-end high-speed detection, making it applicable to a variety of real-time object detection applications (Alif and Hussain, 2025; Tian et al., 2025). To address the high computational complexity of vanilla attention in high-resolution feature modeling, YOLOv12 introduces an area attention module. This module abandons the complex window slicing operations and instead partitions feature maps along vertical or horizontal regions. While maintaining a large receptive field, it reduces the computational complexity of vanilla attention from $2n^{2}hd$ to $\frac{1}{2}n^{2}hd$ (Guo et al., 2025). At the network structure level, YOLOv12 proposes the residual efficient layer aggregation network. This structure builds upon the original efficient layer aggregation networks by integrating residual connections with scaling factors and organizing the feature aggregation process into a bottleneck design. This approach improves model stability and convergence efficiency while effectively reducing computational cost (Sikdar et al., 2025). In addition, YOLOv12 strikes optimization measures such as FlashAttention and delete location coding, achieves a good balance between detection accuracy and speed.

**Figure 3:**
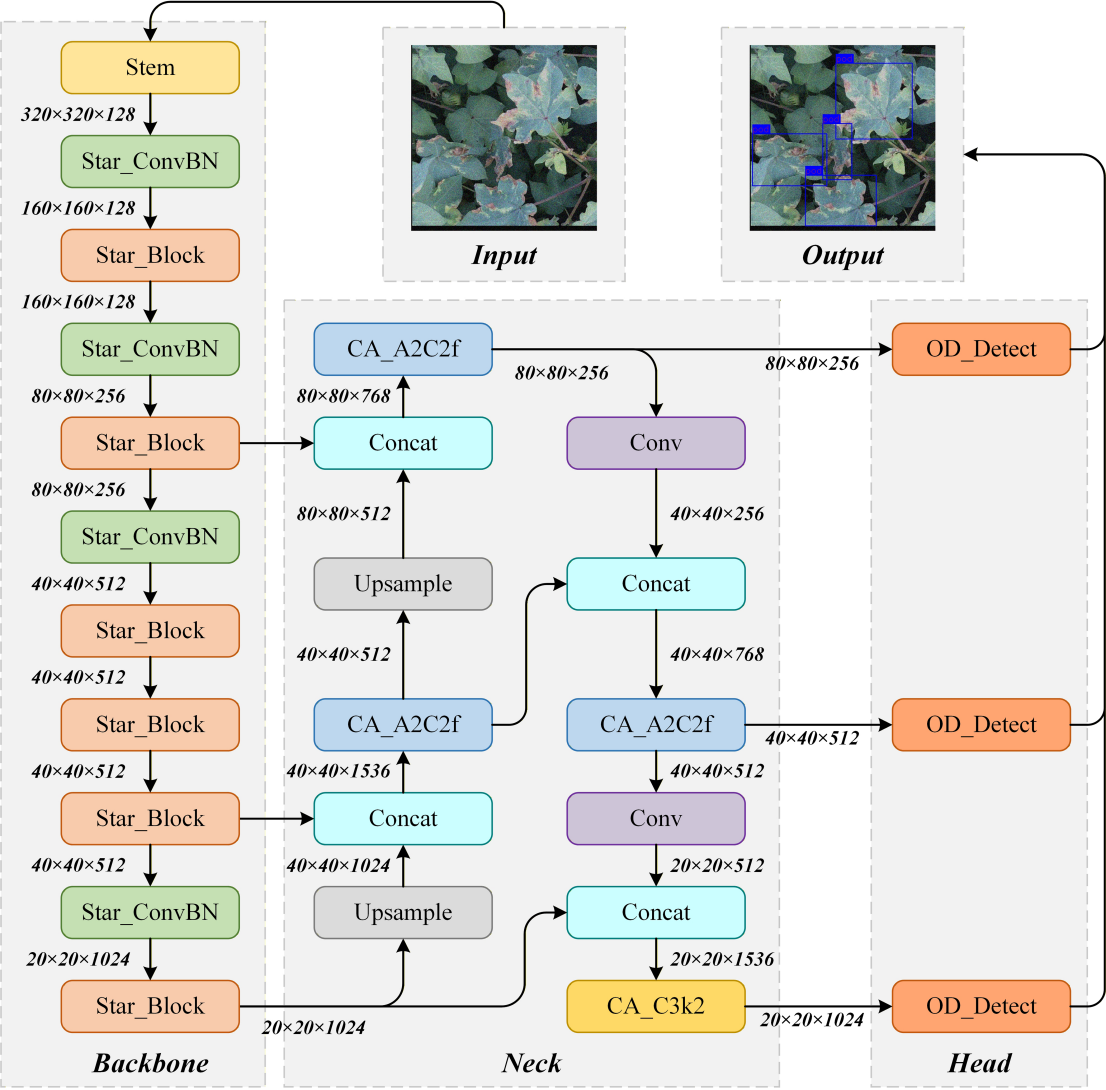
Structure of the YOLO-SCOD model.

This study proposes an enhanced cotton Verticillium wilt detection model YOLO-SCOD based on the YOLOv12 framework, as illustrated in **Figure 3**. The model improves the detection accuracy while effectively controlling the complexity of the model by jointly optimizing the detection head, the neck network’s C3k module, and the backbone network. In the backbone network, StarNet architecture is introduced to enhance multi-level feature modeling. StarNet strengthens the feature representation with a relatively low computing cost through its star operation, so that it can more accurately extract texture and edge information from cotton leaf disease spots. By incorporating the CA block from the multi-order gated aggregation network (MogaNet), the C3k module in the neck network is enhanced. The architecture promotes the interaction and integration between multi-scale features through adaptive channel aggregation. It enhances the model’s ability to represent complex backgrounds and multi-scale disease spots, while maintaining a controllable number of parameters. In the detection head, ODConv replaces the original depthwise convolution (DWConv). ODConv dynamically generates convolutional weights through the multi-dimensional attention mechanism, which effectively improves the positioning and identification accuracy of the model for multi-scale and irregular lesions. Compared with DWConv, ODConv shows better flexibility in feature representation and spatial adaptability. Together, StarNet, CA block, and ODConv form a complementary synergy across feature extraction, feature fusion, and target discrimination. This enables YOLO-SCOD to achieve high detection accuracy and stability in complex environments.

#### 2.2.2 Integration of StarNet in the backbone network

StarNet enhances the nonlinear relationships between features through element-by-element multiplication, thus effectively expanding the feature representation without increasing the channel width (Ma et al., 2024; Shen et al., 2025; Wang et al., 2025a). In this study, StarNet was integrated into the YOLOv12 backbone network and reconstructed the original feature extraction layer to enhance the depth and accuracy of the model in extracting the characteristics of the focal area.

**Figure 4:**
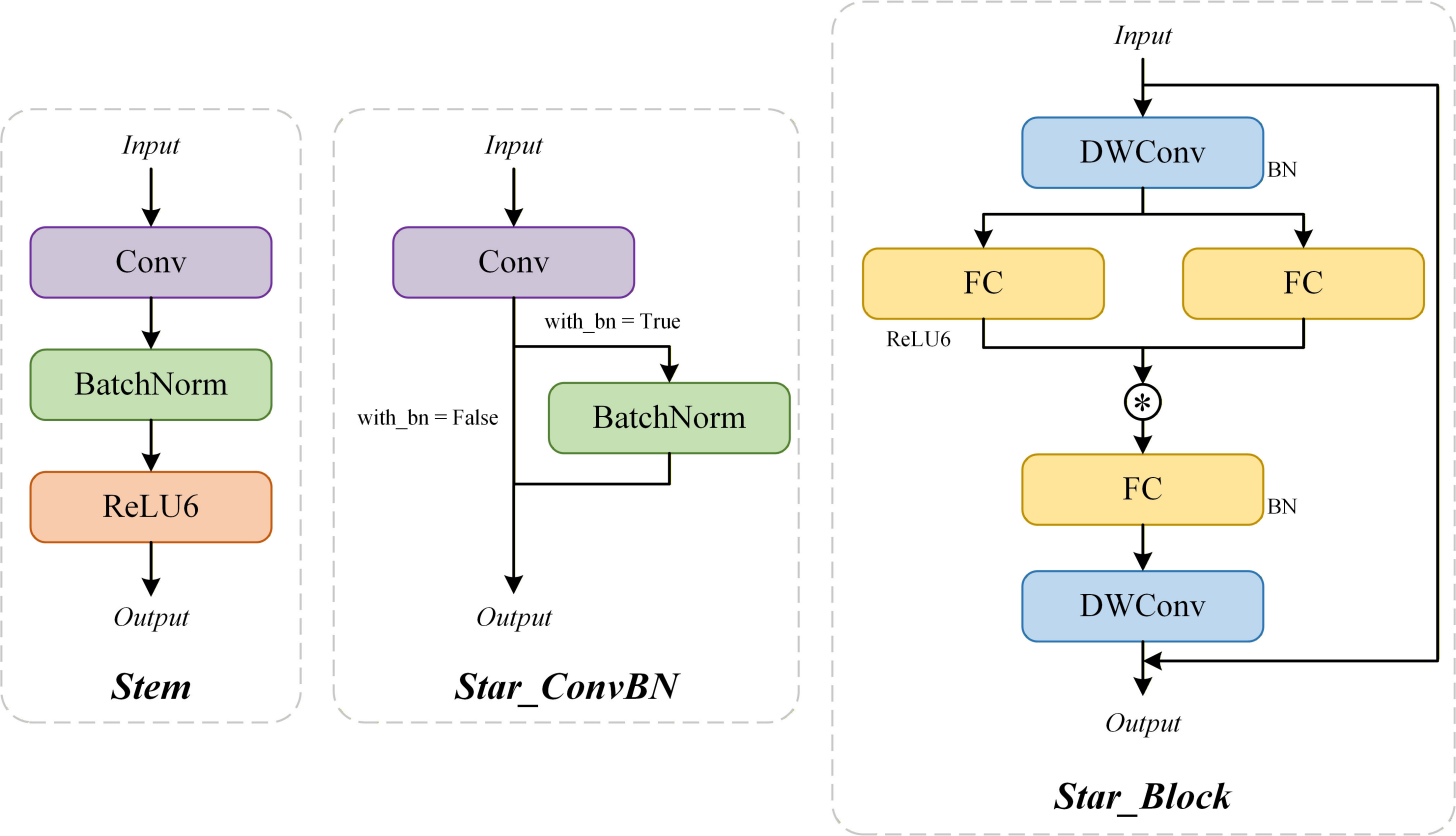
Structure of Stem, Star_ConvBN, and Star_Block in the StarNet backbone network.

The StarNet architecture adopts a hierarchical five-stage network structure to realize multi-scale feature extraction and deep semantic representation. In the initial stage, the Stem module performs preliminary downsampling and feature mapping of the input image. In the second stage, combine Star_ConvBN and Star_Block modules to extract shallow features and capture low-level visual information such as textures and edges. In the third stage, the network further downsamples the feature maps through the Star_ConvBN module and introduces the Star_Block module to effectively expand the sensing field while retaining the key local details. In the fourth stage, multiple Star_Block modules are continuously stacked to deepen the depth of the network and capture more complex advanced semantic features. Meanwhile, a random depth-based regularization strategy is introduced to mitigate overfitting risks during deep network training. During the fifth and concluding phase, the network employs Star_ConvBN together with the final Star_Block to achieve deeper fusion of abstract feature maps. This process enhances the consolidation of overall contextual semantics and strengthens the expressive capability of the model, thereby offering more reliable representations for detecting large-sized targets. The detailed structures of Stem, Star_ConvBN, and Star_Block are illustrated in **Figure 4**, and the mathematical formulation of the star operation within Star_Block is presented in (Equations 1, 2).

(1) $$w_{1}^{T}x*w_{2}^{T}x=\sum_{i=1}^{d+1}\sum_{j=1}^{d+1}w_{1}^{i}w_{2}^{j}x^{i}x^{j}=\frac{\alpha_{\left(1,1\right)}x^{1}x^{1}+\cdots +\alpha_{\left(d+1,d+1\right)}x^{d+1}x^{d+1}}{\left(d+2\right)\left(d+1\right)/2}$$

(2) $$\alpha_{\left(i,j\right)}=\{\begin{array}{l} w_{1}^{i}w_{2}^{j},\;if\;i==j\\ w_{1}^{i}w_{2}^{j}+w_{1}^{j}w_{2}^{i},\;if\;i!=j \end{array}$$

where *i* denotes the spatial index of the feature map, *j* denotes the channel index, *d* denotes the number of input channels, and α is the coefficient for each item.

#### 2.2.3 Integration of the channel aggregation block into the neck network

**Figure 5:**
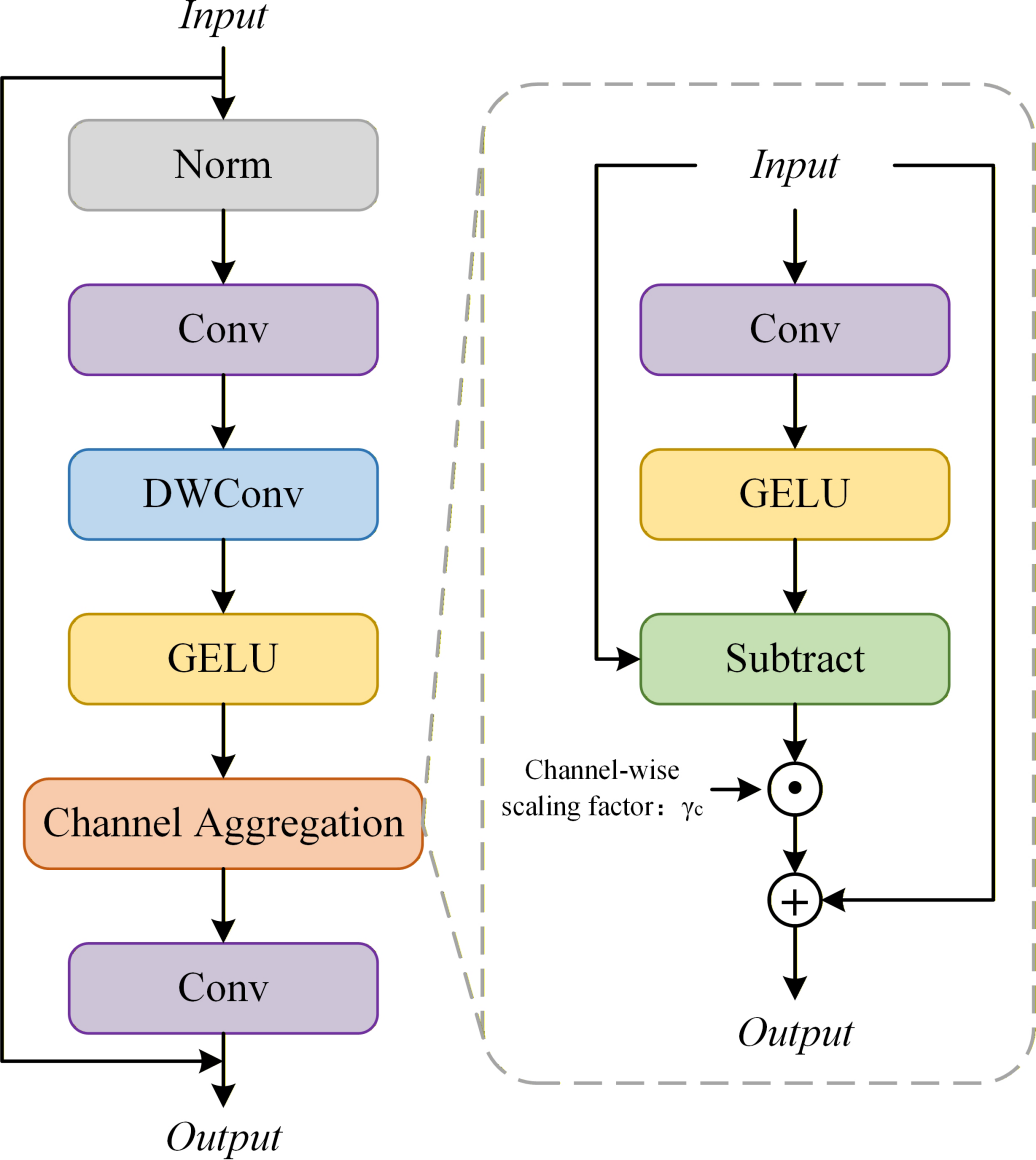
Structure of the channel aggregation block.

**Figure 6:**
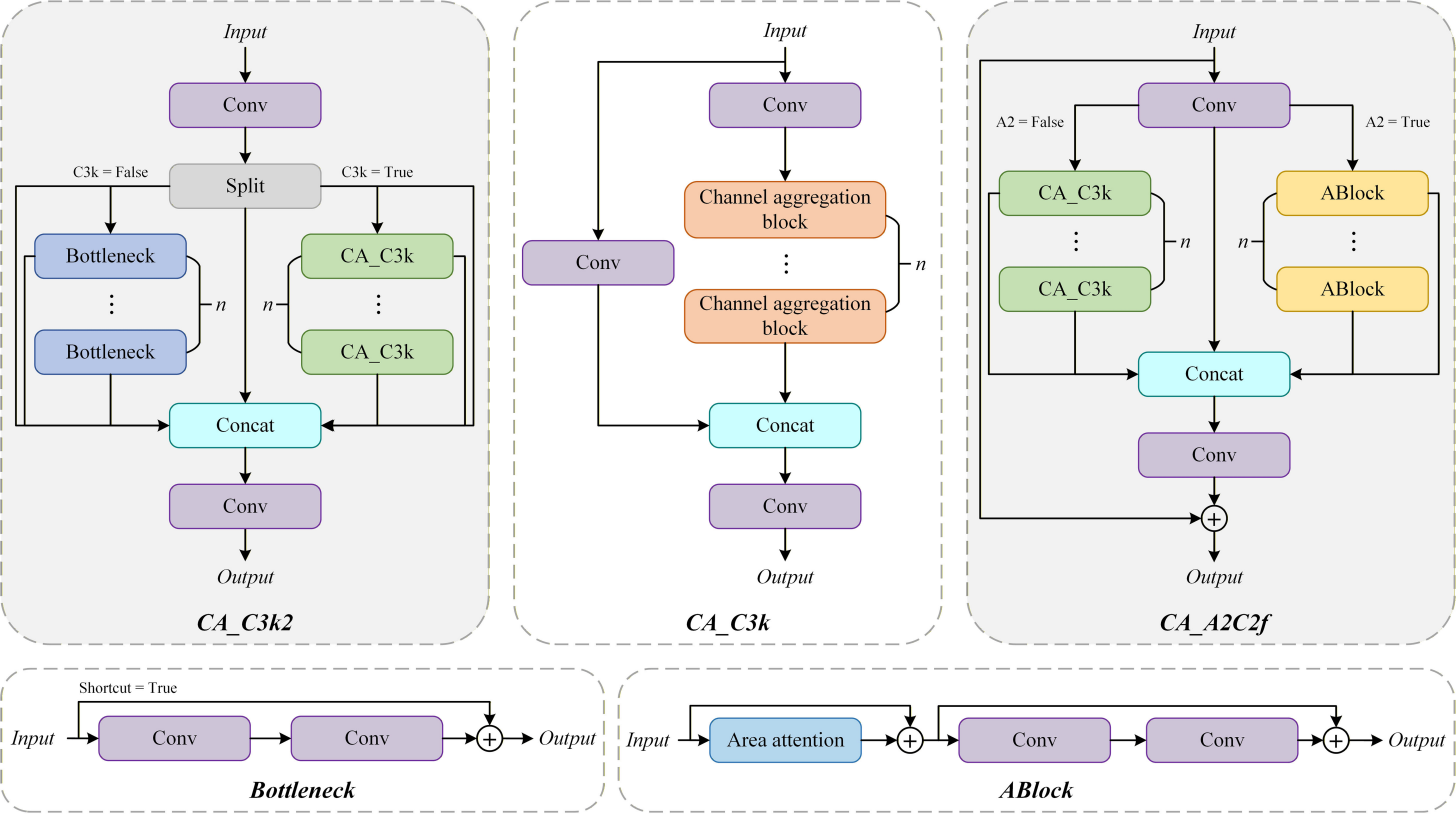
Structure of the CA_C3k2 and CA_A2C2f modules.

In the YOLOv12 framework, the neck network is a key component that directly influences detection performance by facilitating multi-scale feature fusion and cross-layer information propagation. To further enhance the discriminative power and effectiveness of feature representations, this study integrates the CA block into the C3k structures of both the C3k2 and A2C2f modules. **Figure 5** presents the structural design of the CA block, while **Figure 6** depicts the configurations of the optimized C3k2 and A2C2f modules.

The CA block, derived from the MogaNet architecture, is a channel-wise feature enhancement module designed to optimize feature distributions through channel-level aggregation and adaptive reallocation (Li et al., 2022b). By adopting DWConv, the CA block achieves efficient local feature aggregation while maintaining low computational overhead. Furthermore, the module combines channel decomposition with residual weighting strategy and introduces channel-level scaling factors to adaptively adjust the response strength of each channel. This mechanism effectively inhibits redundant information while enhancing information-rich and task-related characteristics. Therefore, the enhanced CA_C3k2 and CA_A2C2f modules show better feature modeling ability, showing higher stability and robustness when dealing with complex textures and fine-grained local disease features. The mathematical formulation of the CA block is provided in (Equation 3).

(3) $$CA\left(X\right)=X+\gamma_{c}⊙\left(X-GELU\left(XW_{r}\right)\right)$$

where $\gamma_{c}$ denotes the channel-wise scaling factor, X denotes the input features, and $W_{r}$ denotes the channel-reducing projection.

#### 2.2.4 Omni-dimensional dynamic convolution module

**Figure 7:**
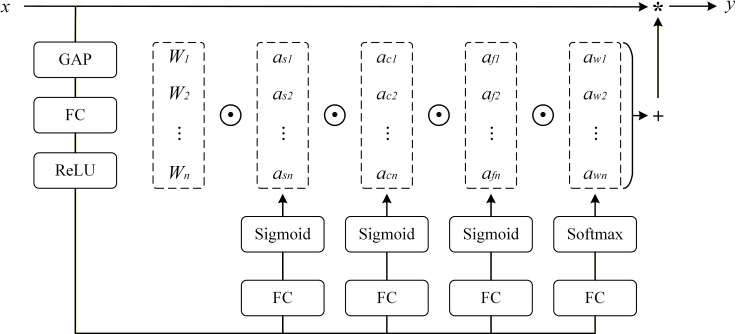
Schematic illustration of the ODConv module.

In order to solve the limitations of DWConv in the inter-channel information interaction and feature representation ability in the YOLOv12 detection head, this study introduces ODConv for structural optimization, as illustrated in **Figure 7**. Unlike the traditional convolutional operation using a fixed convolutional kernel, ODConv introduces a parallel attention mechanism in the four dimensions of spatial, input channel, output channel, and convolution kernel. This design can dynamically and adaptively adjust the convolution parameters (Li et al., 2022a; Zhao et al., 2025). As formulated in (Equation 4), ODConv jointly leverages spatial attention ( $\alpha_{si}$), input channel attention ( $\alpha_{ci}$), output channel attention ( $\alpha_{fi}$), and convolution kernel attention ( $\alpha_{wi}$) to act on the base convolution kernel parameters. By dynamically adjusting the convolution operation in response to the semantic information of the input features, ODConv significantly enhances the flexibility, expressiveness, and adaptability of feature representations, thereby improving the detection head’s capacity for precise localization and accurate recognition.

(4) $$y=\left(\alpha_{w1}⊙\alpha_{f1}⊙\alpha_{c1}⊙\alpha_{s1}⊙W_{1}+\cdots +\alpha_{wn}⊙\alpha_{fn}⊙\alpha_{cn}⊙\alpha_{sn}⊙W_{n}\right)*x$$

where ⊙ denotes the multiplication operation across different dimensions of the kernel space, and * denotes the convolution operation.

**Figure 8:**
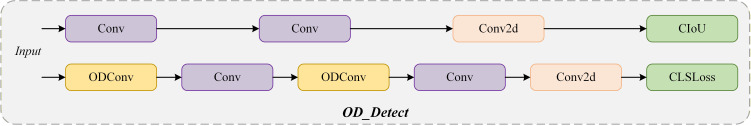
Structure of the OD_Detect module.

**Figure 8** shows the optimized OD_Detect module’s architecture. The OD_Detect module is capable of adaptively adjusting convolution kernel weights in response to features across different scales and semantic levels. The model is better able to concentrate on important target areas by combining spatial attention mechanisms with multi-dimensional weighting techniques. The model’s capacity to classify and precisely locate targets in complicated settings is improved by this architecture.

### 2.3 Experimental setup

All experiments were performed in a consistent hardware and software environment in order to assess the efficacy of the proposed YOLO-SCOD model for identifying Verticillium wilt in cotton. The detailed experimental configuration and training parameter settings are summarized in **Table 1**. Moreover, to comprehensively assess the model’s detection performance in identifying cotton Verticillium wilt lesions, precision (P), recall (R), mAP50, and mAP50–95 were employed as evaluation metrics. The mathematical definitions of these metrics are provided in (Equations 5–8). Metrics for evaluating model computational complexity include: the number of parameters (Params), floating-point operations (FLOPs), and model size. Additionally, frames per second (FPS) is introduced as a metric for evaluating model inference speed.

**Table 1:** Experimental configuration and training parameter settings.

Type	Parameter	Configuration
Environment	CPU	Intel(R) Xeon(R) E5–2680 v4 @ 2.40GHz
	GPU	NVIDIA RTX A2000
	operating system	Ubuntu20.04
	Python	3.10
	PyTorch	2.1.1
	CUDA	11.8
Training	epochs	300
	initial learning rate	0.01
	final learning rate	0.01
	batch size	16
	workers	8
	random seed	0
	patience	100
	optimizer	SGD
	optimizer weight decay	0.0005
	momentum	0.937

(5) $$Precision=\frac{TP}{TP+FP}$$

(6) $$Recall=\frac{TP}{TP+FN}$$

(7) $${mAP}_{50}=\frac{1}{n}\sum_{i=1}^{n}AP_{i}$$

(8) $${mAP}_{50-95}=\frac{1}{n}\sum_{i=1}^{n}\frac{1}{10}\sum_{j=1}^{10}AP_{i,j}$$

where *TP* represents the count of lesion instances that are accurately identified, whereas *FP* refers to the cases incorrectly classified as lesions. *FN* indicates the number of lesion samples that remain undetected, and *n* corresponds to the total number of target categories involved in the detection task. $AP_{i}$ represents the average precision of category *i* at an intersection over union (IoU) of 0.5, and $AP_{i,j}$ represents the average precision of category *i* at an IoU of 0.5+0.05×(j−1).

## 3 Results and discussion

### 3.1 Performance comparison before and after model optimization

**Table 2** presents a comparative evaluation between the proposed YOLO-SCOD framework and the original YOLOv12n model on the cotton Verticillium wilt dataset, considering detection accuracy, computational complexity, and inference speed. The findings demonstrate that the improved model reduces parameter burden while consistently surpassing the baseline across all assessed indicators. Specifically, YOLO-SCOD achieves a P of 0.960 and a R of 0.911, corresponding to increases of 2.019% and 0.220%, respectively, relative to YOLOv12n. Notably, the mAP50–95 is increased to 0.860, corresponding to an improvement of 6.436%, indicating that the enhanced model exhibits improved lesion localization and recognition capability under complex environmental conditions. Regarding computational efficiency, YOLO-SCOD model demonstrates enhanced parameter reduction. Params and FLOPs are reduced to 2.206 M and 5.0 G, respectively, representing decreases of 13.728% and 20.635% compared to YOLOv12n, while the model size is reduced by 12.727%. In addition, YOLO-SCOD demonstrates a certain improvement in inference speed, with a 4.167% increase compared to YOLOv12n.

**Table 2:** Comparison of detection results between the baseline model and the enhanced version.

Model	P	R	mAP50	mAP50-95	Params (M)	FLOPs (G)	Model size (MB)	FPS
YOLOv12n	0.941	0.909	0.967	0.808	2.557	6.3	5.5	96
YOLO-SCOD	0.960	0.911	0.968	0.860	2.206	5.0	4.8	100

**Figure 9:**
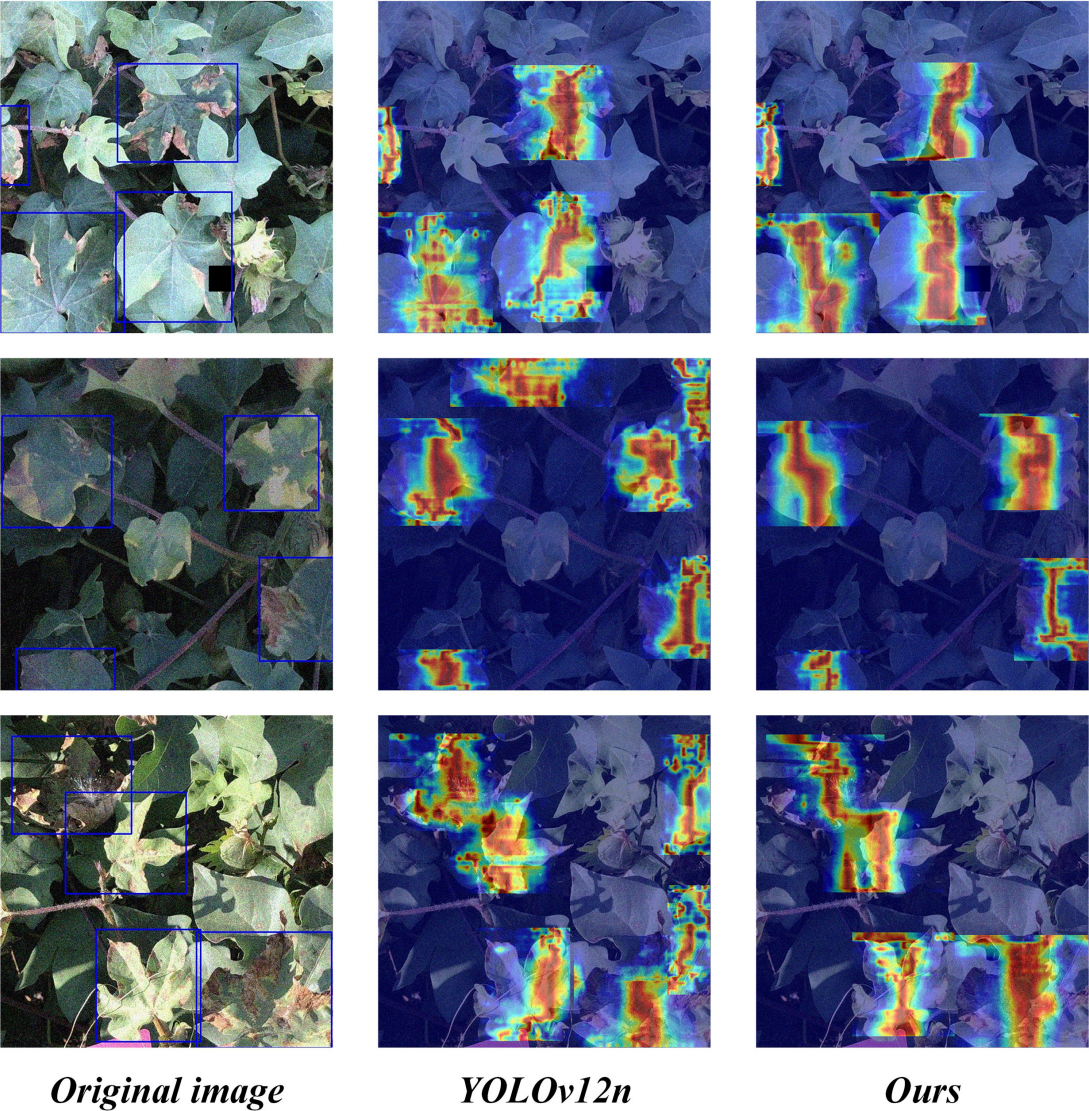
Comparison of heatmaps generated by YOLOv12n and YOLO-SCOD.

**Figure 10:**
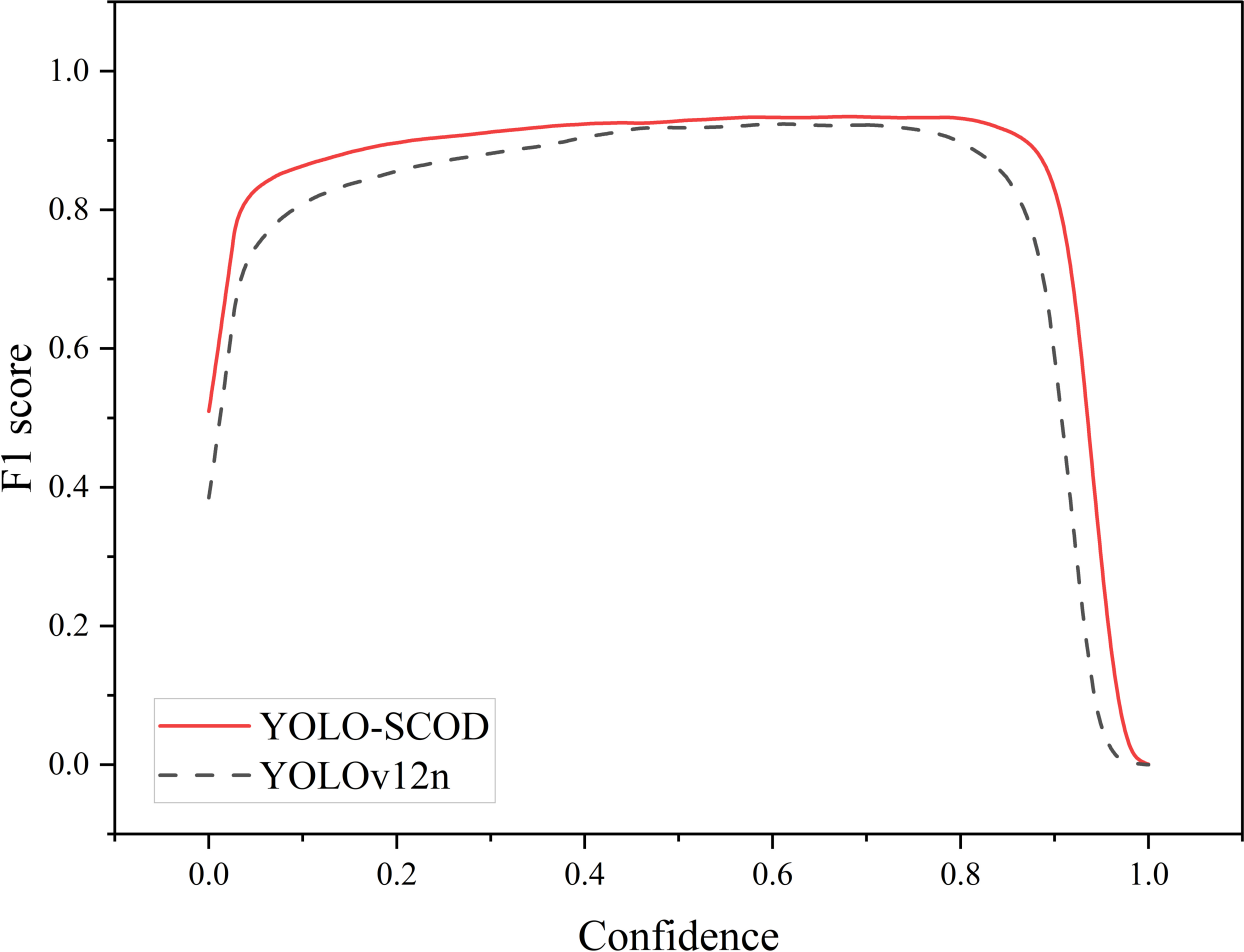
Comparison of F1-confidence curves for YOLOv12n and YOLO-SCOD.

**Figure 9** illustrates the contrastive visualization of feature heatmaps obtained from the original model and the improved version, where red regions indicate key discriminative areas during prediction. The heatmap distribution of YOLOv12n is relatively dispersed and exhibits a certain degree of false activation in background regions. By comparison, the enhanced YOLO-SCOD model generates more focused activations concentrated on Verticillium wilt lesion regions, while effectively mitigating irrelevant background signals, thereby facilitating more precise localization of diseased areas. **Figure 10** shows the F1 value-confidence curve of YOLO-SCOD and YOLOv12n. Both models show a tendency for the F1 value to rise and then decrease with the increase of the confidence threshold, but YOLO-SCOD always maintains a high F1 value in the whole confidence range. At the low confidence level, the F1 value of YOLO-SCOD is significantly higher than that of YOLOv12n, indicating that it can effectively reduce false detections while maintaining a high R value when dealing with weak focal characteristics, thus showing stronger feature distinction ability. Within the confidence interval of 0.2 to 0.8, the F1 score curve of YOLO-SCOD remains more stable and attains higher values, reflecting a more favorable balance between P and R. At high confidence levels, the performance degradation of YOLO-SCOD is relatively delayed and continues to outperform YOLOv12n, suggesting higher reliability of the predicted results and an effective reduction in the erroneous suppression of valid detection bounding boxes. Overall, the YOLO-SCOD model not only effectively improves detection accuracy but also further reduces computational complexity, resulting in superior overall detection performance.

### 3.2 Ablation experiment

To evaluate the specific impact of each component within the YOLO-SCOD framework, ablation experiments with single-module configurations were performed, as summarized in **Table 3**.

**Table 3:** Results of single-module ablation experiments based on YOLOv12n.

StarNet	CA block	OD_Detect	P	R	mAP50	mAP50-95	Params (M)	FLOPs (G)	Model size (MB)	FPS
–	–	–	0.941	0.909	0.967	0.808	2.557	6.3	5.5	96
✓	–	–	0.958	0.923	0.968	0.872	2.339	5.3	5.0	126
–	✓	–	0.941	0.925	0.972	0.837	2.385	6.0	5.2	91
–	–	✓	0.953	0.910	0.969	0.819	2.596	6.3	5.7	88

The results of the single-module ablation experiments demonstrate that introducing the StarNet architecture into the backbone of YOLOv12n effectively improves both detection performance and model complexity. Specifically, P and R increased by 1.807% and 1.540%, respectively, while mAP50–95 is significantly enhanced from 0.808 to 0.872, corresponding to a gain of 7.921%. At the same time, Params, FLOPs, and model size decrease by 8.538%, 15.873%, and 9.091%, respectively, while FPS increases by 31.250%. The StarNet architecture employs a lightweight star operation and an efficient feature mapping strategy, which effectively compress redundant computational overhead while enhancing the network’s capability to represent complex nonlinear features. As a result, the model achieves improved representation of target features under complex background conditions.

When CA_C3k2 and CA_A2C2f are introduced into the neck network of YOLOv12n, mAP50–95 is increased to 0.837, while Params, FLOPs, and model size were reduced to 2.385 M, 6.0 G, and 5.2 MB, respectively, with a slight decrease in FPS of 5.208%. These results indicate that the CA block enables the model to adaptively adjust features in both spatial and channel dimensions, enhancing the network’s focus on critical information during the feature fusion process, thereby improving the stability and discriminative power of feature representations. However, as the CA block employs cross-channel information aggregation and redistribution operations, it reduces computational parallelism to a certain extent, leading to a slight decrease in FPS. In addition, incorporating ODConv into the detection head of YOLOv12n can increase the P by 1.275% and the mAP50–95 by 1.361%, further improving overall detection accuracy. However, Params and model size increased slightly, and FPS decreased by 8.333%. By introducing multi-dimensional attention mechanisms into the convolution operation, ODConv enables dynamic adjustment of convolution weights based on input features, thus enhancing the modeling ability of the detection head to complex targets and improving its adaptability to targets of different shapes and scales. However, ODConv requires the generation of additional attention branches to produce multi-dimensional weights and dynamically reconstructs convolution kernels during inference. This increases computational complexity and memory access costs to a certain extent, resulting in a reduction in the model’s FPS.

Based on the single-module ablation experiment, this study further verifies the impact of combined modules on the performance of YOLOv12n, with the results summarized in **Table 4**. Compared to the original YOLOv12n, integrating the CA block and ODConv modules into both the neck and detection head increased P and mAP50–95 by 1.700% and 4.455%, respectively, while reducing Params and FLOPs by 5.190% and 4.762%, respectively. These results show that the synergistic incorporation of CA block and OD_Detect enhances detection performance without compromising the model’s lightweight design.

**Table 4:** Results of multi-module ablation experiments based on YOLOv12n.

StarNet	CA block	OD_Detect	P	R	mAP50	mAP50-95	Params (M)	FLOPs (G)	Model Size (MB)	FPS
–	–	–	0.941	0.909	0.967	0.808	2.557	6.3	5.5	96
–	✓	✓	0.957	0.941	0.974	0.844	2.424	6.0	5.3	83
✓	✓	–	0.957	0.899	0.964	0.859	2.167	5.0	4.7	115
✓	–	✓	0.958	0.915	0.966	0.868	2.378	5.3	5.1	101
✓	✓	✓	0.960	0.911	0.968	0.860	2.206	5.0	4.8	100

For the YOLOv12n variant using StarNet as the backbone network, the further integration of CA block or OD_Detect improves mAP50–95 by 6.312% and 7.426% respectively, while effectively compressing Params, FLOPs, and model size, and improving FPS by 19.792% and 5.208%, respectively. This observation indicates that even with a lightweight backbone architecture, the feature enhancement in the neck network and the dynamic modeling mechanisms in the detection head can still improve the overall performance of the model. Finally, when the three optimization strategies are applied at the same time, the model achieves the best overall performance, with P reaching 0.960 and mAP50–95 reaching 0.860. At the same time, Params, FLOPs, and model size were reduced to 2.206 M, 5.0 G, and 4.8 MB, respectively, and the FPS increased to 100. Compared with the configuration that simultaneously incorporates the CA block and the OD_Detect, YOLO-SCOD shows slight decreases in R and mAP50, but exhibits more pronounced advantages in terms of computational complexity and inference speed. In addition, although the mAP50–95 of YOLO-SCOD (0.860) is slightly lower than that achieved when both StarNet and OD_Detect are introduced (0.868), the difference is marginal, and the model achieves further improvement in lightweight design. Considering detection accuracy, computational complexity, and inference speed comprehensively, YOLO-SCOD effectively reduces computational cost while maintaining high detection performance, thereby achieving a better balance between accuracy and efficiency.

### 3.3 Comparative experiments

In order to make a comprehensive evaluation of the proposed YOLO-SCOD model, this study conducted a comparative experiment using the same data set conditions in a unified experimental environment. Faster R-CNN, SSD, YOLOv5n, YOLOv6n, YOLOv8n, YOLOv10n, and YOLOv11n were employed as benchmark methods, and the detailed performance results are presented in **Table 5**. Compared with other models in the YOLO series, YOLO-SCOD has excellent performance in various indicators, and its P values and R values are 0.960 and 0.911 respectively, indicating that it has excellent performance in reducing false alarms and missed alarms. In addition, the mAP50–95 of YOLO-SCOD reached 0.860, which is 6.304% and 7.635% higher than YOLOv10n and YOLOv11n respectively, and more than the improvement of YOLOv5n, YOLOv6n and YOLOv8n. The research results show that the proposed method maintains consistent and accurate detection ability within a wide range of IoU threshold settings. In terms of inference speed, YOLO-SCOD achieves performance comparable to YOLOv10n and YOLOv11n; while it is slightly slower than YOLOv5n, YOLOv6n, and YOLOv8n, it significantly outperforms Faster R-CNN and SSD.

**Table 5:** Comparison of experimental results for different object detection models.

Model	P	R	mAP50	mAP50-95	Params (M)	FLOPs (G)	Model size (MB)	FPS
Faster R-CNN	0.882	0.948	0.895	0.701	41.352	216.0	157.7	23
SSD	0.910	0.925	0.934	0.650	23.746	60.9	95.0	50
YOLOv5n	0.884	0.883	0.946	0.765	2.503	7.1	5.3	125
YOLOv6n	0.900	0.875	0.946	0.767	4.234	11.8	8.7	122
YOLOv8n	0.915	0.876	0.947	0.771	3.006	8.1	6.3	133
YOLOv10n	0.924	0.889	0.959	0.809	2.695	8.2	5.8	102
YOLOv11n	0.942	0.906	0.966	0.799	2.582	6.3	5.5	103
YOLO-SCOD	0.960	0.911	0.968	0.860	2.206	5.0	4.8	100

In terms of model complexity, YOLO-SCOD exhibits a more compact architecture without compromising detection precision. Its Params and FLOPs are only 2.206 M and 5.0 G, respectively, which are lower than other YOLO models. Although compared with the Faster R-CNN and SSD models, the R of YOLO-SCOD is reduced by 0.037 and 0.014 respectively, its P, mAP50, mAP50-95, and inference speed have all seen significant improvements, while model complexity has been substantially reduced. Through the coordinated optimization of the backbone network, neck structure and detection head, YOLO-SCOD enhances feature representation capability while effectively avoiding an increase in model complexity, thereby achieving a balance between detection accuracy and computational cost.

### 3.4 Limitation analysis

Although the YOLO-SCOD model provides an efficient solution for the intelligent identification of cotton Verticillium wilt, it still has certain limitations. Compared with the YOLOv12n model, the improvements achieved by YOLO-SCOD in R and mAP50 are relatively limited, and the overall detection accuracy still requires further enhancement. Furthermore, the current dataset is insufficient in terms of regional coverage and environmental diversity, and its scale needs to be further expanded. Moreover, the present study does not include grouped testing or comparative analysis under different growth stages, varying levels of occlusion, or diverse complex background conditions. In addition, this study is still at the stage of model development and offline validation, and no deployment or field-based experimental verification has yet been conducted. Therefore, the practical performance, stability, and generalization ability of the model in real-world agricultural scenarios still need to be further evaluated and optimized.

## 4 Conclusions

This study proposes YOLO-SCOD, an improved YOLOv12n-based detection model for cotton Verticillium wilt, which can efficiently and automatically identify disease spots in complex field environments. By integrating StarNet, the CA block, and ODConv, the model introduces structural optimization at multiple network levels, thus improving the overall detection performance. The main conclusions are as follows:

1. The StarNet architecture incorporated into the backbone network enables high-order nonlinear reconstruction of cross-channel features through its star-shaped feature mapping mechanism. This design improves feature representation capability while maintaining a lightweight structure, thereby enhancing the model’s ability to capture multi-scale targets.
2. The CA block embedded within the C3k module of the neck network aggregates channel-wise features and adaptively assigns weights, improving the model’s ability to focus on critical lesion features and enhancing the discriminative power of feature fusion. However, this improvement comes at the cost of a slight reduction in inference speed.
3. The integration of ODConv into the detection head allows convolutional kernel weights to be dynamically adjusted across multiple dimensions, effectively overcoming the limitations of DWConv in channel interaction. This leads to improved detection performance in complex environments. Nevertheless, its contribution to model compression is relatively limited, and it introduces additional computational overhead, resulting in reduced inference speed.
4. The final YOLO-SCOD model demonstrates strong performance in both detection accuracy and model lightweighting for cotton Verticillium wilt identification. Compared with YOLOv12n, YOLO-SCOD achieves P and R of 0.960 and 0.911, respectively, with an increase of 6.436% in mAP50-95. At the same time, Params and FLOPs were reduced to 2.206 M and 5.0 G respectively, which was reduced by 13.728% and 20.635% respectively, relative to YOLOv12n. In addition, the detection speed is increased by 4.167%, further demonstrating the advantages of YOLO-SCOD in lightweight design.

Future work will focus on further expanding the scale of the cotton Verticillium wilt image dataset and constructing a more diverse dataset that covers multiple regions, environmental conditions, and growth stages. Furthermore, grouped validation will be conducted under varying lighting conditions, degrees of occlusion, and growth stages to systematically analyze the model’s detection performance under complex and extreme conditions, thereby further improving its generalization ability and robustness. In addition, more advanced deep learning architectures and optimization strategies will be incorporated to further refine the model. Field deployment and practical application experiments will also be conducted to validate the model’s performance in real-world scenarios, providing more reliable technical support for the intelligent detection and precise management of cotton Verticillium wilt.

## Data Availability

The raw data supporting the conclusions of this article will be made available by the authors, without undue reservation.
